# Contemporary trends and outcomes in European ICD recipients: a 15-year analysis

**DOI:** 10.1093/europace/euag110

**Published:** 2026-06-10

**Authors:** Andrea Papa, Salvatore Chianese, Sing-Chien Yap, Michael Kühne, Christian Sticherling, Felix Mahfoud, Dominic A M J Theuns, Beat Schaer

**Affiliations:** Department of Cardiology, University Hospital Basel, Petersgraben 4, 4031 Basel, Switzerland; Cardiovascular Research Institute Basel, Basel, Switzerland; Cardiology Division, A. Cardarelli Hospital, Naples, Italy; Department of Advanced Biomedical Sciences, “Federico II” University, Naples, Italy; Department of Cardiology, Cardiovascular Institute, Erasmus MC, Rotterdam, The Netherlands; Department of Cardiology, University Hospital Basel, Petersgraben 4, 4031 Basel, Switzerland; Cardiovascular Research Institute Basel, Basel, Switzerland; Department of Cardiology, University Hospital Basel, Petersgraben 4, 4031 Basel, Switzerland; Cardiovascular Research Institute Basel, Basel, Switzerland; Department of Cardiology, University Hospital Basel, Petersgraben 4, 4031 Basel, Switzerland; Cardiovascular Research Institute Basel, Basel, Switzerland; Department of Cardiology, Cardiovascular Institute, Erasmus MC, Rotterdam, The Netherlands; Department of Cardiology, University Hospital Basel, Petersgraben 4, 4031 Basel, Switzerland; Cardiovascular Research Institute Basel, Basel, Switzerland

**Keywords:** Implantable cardioverter-defibrillator, Temporal trends, Ventricular arrhythmia, Heart failure, Risk stratification

## Introduction

Implantable cardioverter-defibrillators (ICDs) prevent sudden cardiac death in selected patients with reduced left ventricular ejection fraction (LVEF).^1–5^ Over the past two decades, guideline-directed medical therapy (GDMT) and device management have evolved substantially, potentially lowering ventricular arrhythmia burden and the absolute benefit of prophylactic ICD implantation.^6–9^ Contemporary European data capturing these transitions with adjudicated device therapies remain limited. We assessed 15-year temporal trends in patient profile, ICD therapies, and outcomes in two tertiary European centres and identified predictors of appropriate ICD therapy within 60 months.

## Methods

We included all consecutive patients undergoing *de novo* ICD implantation at Erasmus MC (Rotterdam) and University Hospital Basel between 2002 and 2017, grouped by implantation period (2002–05, 2006–09, 2010–13, 2014–17). Stored electrograms were reviewed by local electrophysiology experts to adjudicate arrhythmic episodes. The primary outcome was first appropriate ICD therapy (ATP and/or shock) for ventricular tachycardia (VT) or ventricular fibrillation (VF), censored at 60 months. Secondary outcomes included death, heart transplantation, and a composite of appropriate ICD therapy/transplant/death. Kaplan–Meier curves compared time groups. Because proportional hazards assumptions were violated for implantation era in time-to-event models, we used multivariable logistic regression for the 60-month ICD therapy endpoint, adjusting for prespecified clinical variables, medications, device type, and time group.

## Results

**Figure 1:**
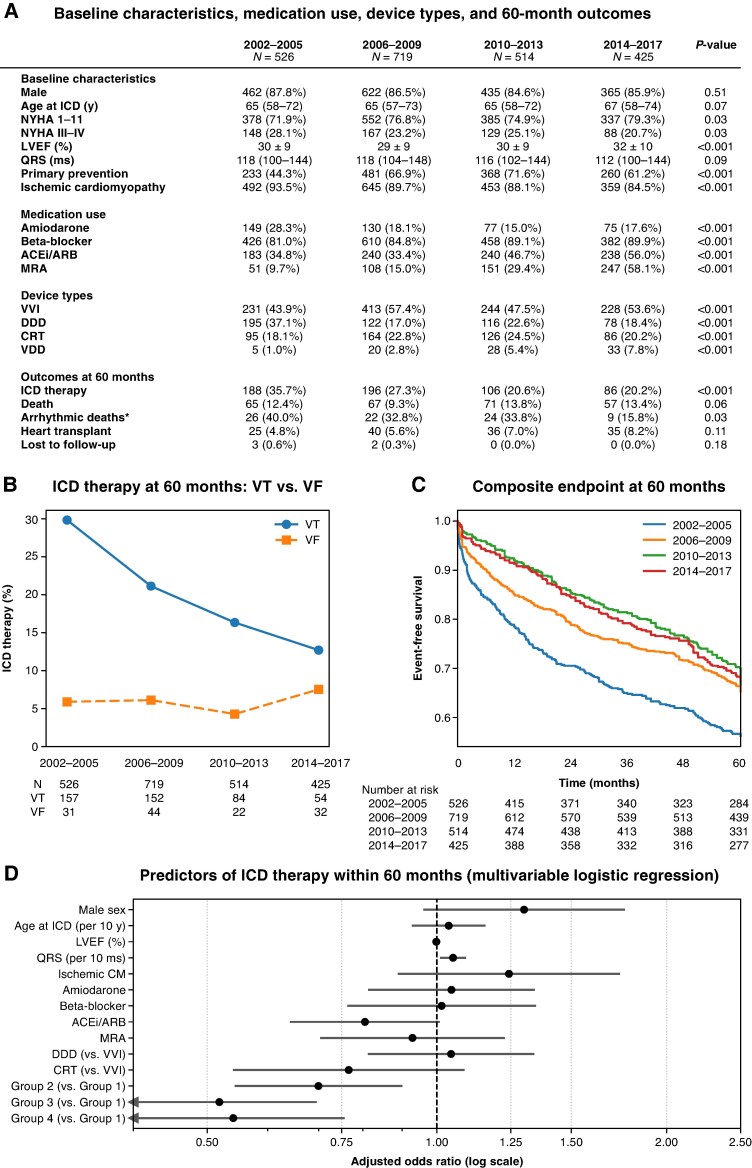
Temporal trends in ICD recipients and outcomes (2002–17). (*A*) Baseline characteristics, medication use, device types, and 60-month outcomes by implantation period (2002–05, 2006–09, 2010–13, 2014–17). (*B*) VT and VF treated by ICD therapy within 60 months (% of each time group); counts for total *N* and VT/VF events are shown below the x-axis. (*C*) Kaplan–Meier curves for the composite endpoint (first appropriate ICD therapy, heart transplantation, or death) to 60 months, with numbers at risk. (*D*) Multivariable logistic regression for appropriate ICD therapy within 60 months (adjusted ORs with 95% CIs; log scale). ACEi/ARB, angiotensin-converting enzyme inhibitor/angiotensin receptor blocker; CI, confidence interval; CRT, cardiac resynchronization therapy; ICD, implantable cardioverter-defibrillator; LVEF, left ventricular ejection fraction; MRA, mineralocorticoid receptor antagonist; NYHA, New York Heart Association; OR, odds ratio; VF, ventricular fibrillation; VT, ventricular tachycardia.

Among 2184 patients (mean age 65.6 years; 13.7% women), 61.4% received ICDs for primary prevention. Across time groups, the proportion of primary-prevention ICDs increased from 44.3% in the earliest period to 61.2% in the most recent period (*Figure 1A*). Over time, recipients had modestly higher LVEF and better functional status, with higher GDMT uptake, and device-type distribution shifted (*Figure 1A*). At 60 months, appropriate ICD therapy occurred in 26.4% overall and declined from 35.7% (2002–05) to 20.2% (2014–17), driven by fewer VT events (29.8–12.7%), whereas VF remained low and did not change materially (*Figure 1B*). Composite event-free survival improved from 56.3 to 67.3% at 60 months (*Figure 1C*). Overall mortality did not differ significantly across eras, but the proportion of deaths classified as arrhythmic decreased (*Figure 1A*). In adjusted analysis, QRS duration independently predicted ICD therapy, whereas LVEF was not independently associated; later implantation eras had lower odds of therapy (*Figure 1D*).

## Discussion

In this dual-centre European cohort spanning 2002–17, we observed a marked decline in adjudicated appropriate ICD therapies within 60 months, driven primarily by fewer sustained VT events, while VF remained low and broadly unchanged. In parallel, composite event-free survival improved across implantation periods. Together, these data suggest that contemporary ICD recipients, selected and treated in current practice, experience a lower treated ventricular arrhythmia burden than recipients implanted in earlier periods.

Several mechanisms likely contributed to the results. First, baseline profile shifted towards less symptomatic heart failure stages and modestly higher LVEF at implantation, consistent with a lower arrhythmic substrate at baseline. Second, uptake of GDMT increased substantially over time, particularly therapies known to reduce malignant ventricular arrhythmias and sudden death risk in heart failure populations. Third, contemporary management of ischaemic heart disease (including broader use of revascularization and secondary prevention) may have reduced ischemia-triggered sustained VT. Fourth, temporal changes in ICD management may have altered the treated arrhythmia burden: contemporary programming strategies (higher rate cut-offs, longer detection times, and optimized discrimination algorithms) reduce therapies for slower or self-terminating VT while preserving detection of VF.^6^ The observed pattern of substantial VT reduction with stable VF fits well with a combination of improved substrate and evolving programming, although programming parameters were not uniformly available for direct testing.

Clinically, declining device therapy rates have two important implications. First, the expected absolute benefit of prophylactic ICD implantation may be smaller in certain contemporary subgroups than in historical, guideline-defining trial populations, raising the practical relevance of individualized benefit assessment and competing-risk profiling.^7^ Second, QRS duration emerged as an independent marker associated with appropriate ICD therapy in the multivariable model, possibly reflecting an adverse electrical substrate (e.g. scar-related slow conduction, fibrosis, electrical remodelling, and dyssynchrony) that may predispose to re-entrant VT. Left ventricular ejection fraction remains central to guideline-based eligibility, but within an already-selected ICD population its ability to further discriminate arrhythmic risk may be limited. This supports ongoing efforts to refine risk stratification using electrical markers and phenotyping beyond systolic function alone, with the goal of improving the ‘signal-to-noise’ ratio of ICD implantation in the modern era. In this context, a more recent ICD implantation remained associated with lower odds of therapy, highlighting that ‘group effects’ are not fully captured by individual baseline variables and likely reflect integrated changes in therapy, selection, and device management.

## Limitations

This study is retrospective and therefore subject to residual confounding, evolving documentation practices, and unmeasured differences in follow-up intensity across eras. Although arrhythmic episodes were adjudicated using stored electrograms, heterogeneity in ICD programming (detection zones, therapy delays, and discrimination settings) could not be fully accounted for and may partially explain temporal changes in treated VT. We censored analyses at 60 months to harmonize follow-up across implantation periods; nevertheless, differential longitudinal follow-up beyond 60 months may influence longer-term trajectories. The cohort ends in 2017 and predates widespread adoption of newer heart failure therapies (e.g. sacubitril/valsartan and sodium–glucose cotransporter 2 inhibitors), which could further reduce contemporary arrhythmic risk; therefore, our estimates may represent a conservative picture of current event rates.^8,9^ Finally, the study included two tertiary centres, which may limit generalizability to other healthcare settings, and temporal shifts in ICD implantation referral and evolution of ischaemic heart disease management may also have contributed to group differences, but granular longitudinal data were not available.

## Conclusions

Across 2002–17, European ICD recipients experienced substantially fewer appropriate ICD therapies, largely driven by fewer VT events, with improved composite event-free survival. These trends highlight the need to recalibrate ICD selection and programming and to advance contemporary risk stratification beyond LVEF.^10^

## Data Availability

De-identified data and analysis code are available from the corresponding author upon reasonable request and subject to institutional data-sharing agreements.
